# Factors for the optimal selection of granulocyte colony-stimulating factor preparations and predictors for R-CHOP dose reductions/delays among patients with non-Hodgkin B-cell lymphoma (STOP FN in NHL 2 subanalysis)

**DOI:** 10.1186/s12885-021-08068-0

**Published:** 2021-04-06

**Authors:** Masahiro Yokoyama, Yoshiharu Kusano, Norihito Inoue, Noriko Nishimura, Yuko Mishima, Tomoyuki Nukada, Kiyohiko Hatake, Yasuhito Terui

**Affiliations:** 1 Division of Hematology Oncology, The Cancer Institute Hospital, Japanese Foundation for Cancer Research, 3-8-31 Ariake, Koto-ku, Tokyo, 135-8550 Japan; 2 Medical Affairs, Kyowa Kirin Co. Ltd, Tokyo, Japan; 3 Department of Hematology, International University of Health and Welfare, Tokyo, Japan

**Keywords:** R-CHOP, Febrile neutropenia, Non-Hodgkin B-cell lymphoma, Risk factor, G-CSF, Elderly

## Abstract

**Background:**

A classification tree was used to analyze background factors for granulocyte colony-stimulating factor (G-CSF) preparation selection for febrile neutropenia (FN) prophylaxis in Japanese patients with non-Hodgkin B-cell lymphoma receiving the first R-CHOP cycle.

**Methods:**

This was a subanalysis of the retrospective observational study STOP FN in NHL 2 (UMIN000029534). Patient characteristics, changes in neutrophil count, incidence and severity of neutropenia, and risk factors for dose reduction/delay of R-CHOP were assessed by G-CSF formulation.

**Results:**

Among 234 patients in cycle 1, 25.6% received no G-CSF preparation, 52.1% received daily G-CSF, and 22.2% received pegfilgrastim. Pegfilgrastim use was most frequent among patients aged ≥ 80 years, while that of daily G-CSF was most frequent in patients with lymphocyte count (LC) < 1000 cells/μL. Changes in neutrophil count were more marked with pegfilgrastim compared with daily G-CSF and no G-CSF. Relevant factors for G-CSF preparation selection in the first R-CHOP cycle were age ≥ 80 years and LC < 1000 cells/μL; for chemotherapy dose reduction were FN onset in cycle 1 and female sex; and for dose delay was hemoglobin (< 12 g/dL). After cycle 2 and onward, pegfilgrastim use increased markedly (72.6%) compared with cycle 1 (22.2%), with significantly greater proportions continuing pegfilgrastim use and switching from daily G-CSF.

**Conclusion:**

Relevant factors for G-CSF preparation selection were age ≥ 80 years and LC < 1000 cells/μL. The use of pegfilgrastim increased markedly after cycle 2. These results may be useful for selecting appropriate G-CSF preparations in the first R-CHOP cycle.

**Trial registration:**

UMIN000029534; registered on 13 October 2017, https://upload.umin.ac.jp/cgi-open-bin/ctr_e/ctr_view.cgi?recptno=R000033733.

**Supplementary Information:**

The online version contains supplementary material available at 10.1186/s12885-021-08068-0.

## Background

Febrile neutropenia (FN) is the most common and severe complication of patients receiving chemotherapy for cancer [1]. Developing FN, especially early during chemotherapy, may negatively impact the patient’s prognosis as it may limit treatment duration and effectiveness [1]. A study of 2692 patients undergoing chemotherapy in a community oncology setting in the US reported that first-cycle dose reductions, in response to both the perceived risk of FN and the actual occurrence of FN, were common (23.6%). Additionally, unplanned delays in initiating subsequent chemotherapy cycles occurred in 22.2% of patients [2]. Unfortunately, reductions in the relative dose intensity (RDI) and number of chemotherapeutic cycles can negatively affect patient survival outcomes [3–5]. However, the risks associated with chemotherapy modification must be balanced by the physician against the risks associated with developing FN, which include more frequent and prolonged hospitalization, and increased mortality [6]. Several risk factors for the development of FN have been recently identified among patients with non-Hodgkin B-cell lymphoma (B-NHL) receiving rituximab, cyclophosphamide, doxorubicin, vincristine, and prednisolone (R-CHOP) chemotherapy [7]. The main risk factors are albumin < 35 g/L or RDI < 85%, and lack of prophylaxis against FN.

In the past decade, the prophylactic use of granulocyte colony-stimulating factor (G-CSF) to mitigate the development of FN has become more frequent and has led to a reduced incidence of FN and a corresponding decrease in the number of patients experiencing chemotherapy dose reductions [8, 9].

We reported that Japanese patients treated with two types of G-CSF preparations, daily G-CSF and pegfilgrastim, had incidences of FN of 7.3 and 3.7%, respectively, while patients who did not receive any G-CSF preparation had an incidence of FN of 23.0% [10]. Clinical practice guidelines in Japan [11] and elsewhere [12–14] recommend the use of G-CSF prophylaxis in patients at high risk of developing FN, especially in those receiving chemotherapy regimens with FN incidence greater than 20%. As the R-CHOP chemotherapy regimen is associated with an estimated FN incidence between 10 and 20%, all patients with risk factors are recommended to receive prophylaxis with G-CSF [15, 16].

Studies have shown that prophylaxis with daily G-CSF or pegfilgrastim can reduce the incidence of FN in patients receiving R-CHOP therapy [9, 17, 18]. However, no published studies have examined the associations between G-CSF administration, outcomes, and baseline characteristics of patients in daily practice. Yet, for patients to receive the maximum benefit from prophylaxis with G-CSF, it is necessary to identify the most suitable recipients.

Furthermore, although it is known that the onset of FN in cycle 1 brings about dose reduction and delay of chemotherapy in subsequent cycles, no previous studies in Japan have reported on risk factors that may cause R-CHOP dose modification. In this study, a classification tree was used to analyze background factors for drug selection by G-CSF preparation in the first R-CHOP cycle. Additionally, we analyzed FN incidence, patterns of G-CSF preparation and identified possible predictors of dose reduction and delay of R-CHOP therapy among Japanese patients with B-NHL receiving chemotherapy in a real-world setting.

## Methods

### Study design

This was a subanalysis of a retrospective observational study conducted between January 2015 and June 2017 at the Cancer Institute Hospital of the Japanese Foundation for Cancer Research, Tokyo, Japan.

Full details of the STOP FN in NHL 2 study (UMIN000029534) have been published [10]. In brief, we used the database of our institute to extract data from the medical records of patients who were treated at our institute and met the eligibility criteria. During cycle 1, all patients were hospitalized. During cycle 2 and subsequent cycles, patients were managed in an outpatient basis and only hospitalized if deemed necessary. The type of prophylactic treatment, daily G-CSF or pegfilgrastim, and the corresponding dosing that the patients received were decided by each patient’s treating physician. Further, in cycle 1, daily G-CSF was started at a mean (standard deviation [SD]) of 10.18 (2.67) days, and pegfilgrastim was started at a mean (SD) of 2.59 (1.39) days. Daily G-CSF was administered for a mean (SD) of 2.98 (1.58) days. In cycle 1, 2.4 and 90.7% of patients receiving daily G-CSF and pegfilgrastim, respectively, started treatment on days 0–3 [10].

The study was approved by the institutional Ethical Review Board. The analysis was conducted in accordance with the Declaration of Helsinki and Ethical Guidelines for Medical Research on Individuals, the Law Concerning the Protection of Personal Information, and all other applicable laws and regulations concerning the handling of personal information. As this was a retrospective observational study on data from medical records, the need for informed consent from patients was waived.

### Patients

Patients diagnosed with B-NHL who had received and completed R-CHOP regimens, or patients who had discontinued the R-CHOP treatment after receiving three or more scheduled cycles were eligible for inclusion in this study. The main exclusion criterion was a diagnosis of human immunodeficiency virus-related B-NHL.

For this subanalysis, patients were divided into three groups according to the type of FN prophylaxis received in cycle 1: those who did not receive any prophylactic G-CSF preparation (no G-CSF administration); those who received daily G-CSF; and those who received FN prophylaxis with 3.6 mg pegfilgrastim by subcutaneous administration.

### Measures

Characteristics of patients prior to the initial R-CHOP regimen were collected for evaluation, including age, sex, performance status (PS), body mass index (BMI), disease characteristics (diagnosis, stage, and presence or absence of myeloid infiltration), presence or absence of complications (including diabetes, liver/kidney/heart disease, and incomplete wounds), presence or absence of previous history (last [< 1 month prior to initiation of (R) CHOP regimens] infection or FN), and hematologic parameters (albumin, total bilirubin, hemoglobin, absolute neutrophil count [ANC], and absolute lymphocyte count).

Data related to each chemotherapy cycle, including details related to the prescribed R-CHOP regimen (i.e., drug doses and days to the next cycle), were collated for analysis. Dose reduction was defined as more than a 20% reduction in the dose of cyclophosphamide or doxorubicin in each cycle after cycle 2 compared with that in the first cycle. Dose delay was defined as more than 7 days behind schedule in each cycle after cycle 2.

Information related to the development of FN after completion of the R-CHOP regimen was also analyzed, including body temperature, neutrophil counts, preventive and therapeutic measures (oral antibiotics, G-CSF, and treatment days calculated from the initiation of chemotherapy), and hospitalizations for the development of FN. For hospitalized patients, temperature measurements were taken daily and prior to initiation of the R-CHOP regimen and blood samples were collected every 2 days, wherever possible. For outpatients, temperature measurement was self-reported by the patient.

### Outcomes

The specific outcomes assessed during the study were: identification of patient background factors that may aid in the selection of G-CSF preparation (pegfilgrastim or daily G-CSF); changes in neutrophil count in cycle 1 by G-CSF preparation; duration (number of days) of neutropenia (< 500 cells/μL), the incidence of grade 4 neutropenia (< 500 cells/μL), lowest neutrophil count (neutrophil nadir), and day of onset of the neutrophil nadir, all by type of G-CSF preparation; identification of risk factors that may cause dose reduction or delay of R-CHOP therapy after cycle 2; and actual status of treatment with pegfilgrastim, daily G-CSF, or no G-CSF after cycle 2.

### Statistical analysis

A target sample size for this analysis was not statistically calculated, and all patients identified in the database who met the study eligibility criteria during the study period were included. A classification tree was used to analyze 15 background factors for drug selection by G-CSF preparation in the first R-CHOP cycle [19]. Supplementary Table 1 (see Additional file 1) shows the analysis setting and variables used to create the classification tree. Two-tailed Dunnett’s multiple comparisons tests were performed with pegfilgrastim as a control group to compare outcomes associated with neutrophil count in cycle 1 by G-CSF type. Univariate and multivariate logistic regression with stepwise selection was used to identify factors associated with dose reduction or delay of anticancer drugs after the second cycle of R-CHOP. All statistical analyses were conducted by the Institute of Japanese Union of Scientists & Engineers, using SAS Version 9.3 (SAS Institute Inc., Cary, NC, USA).

## Results

### Patients

The STOP FN in NHL 2 study [10] enrolled a total of 239 patients. For the current analysis, five patients were excluded because the necessary data were not available. Thus, 234 patients were included: 60 (25.6%) patients did not receive any G-CSF, 122 (52.1%) received daily G-CSF, and 52 (22.2%) received pegfilgrastim in cycle 1.

Table 1 summarizes the patient and disease characteristics before the first cycle of R-CHOP. Overall, 53.0% of patients were women and 47.0% were men. Patients had a mean (SD) age of 63.7 (12.7) years. Slightly more women than men received daily G-CSF and pegfilgrastim. The majority (75.6%) of patients had a PS of 0, and this was similar across the three groups. Most patients (67.5%) were diagnosed with diffuse large B-cell lymphoma, followed by follicular lymphoma (17.1%), and other (9.8%); a similar pattern was observed across all three groups. The mean (SD) ANC at baseline was 4147.7 (1425.1), 4246.5 (2286.0), and 4043.8 (1767.7) cells/μL in the no G-CSF administration, daily G-CSF, and pegfilgrastim groups, respectively.

**Table 1:** Patient and disease characteristics before first cycle of R-CHOP

Characteristic	Overall ***N*** = 234	G-CSF type in cycle 1		
		No G-CSF administration ***n*** = 60	Daily G-CSF ***n*** = 122	Pegfilgrastim ***n*** = 52
Sex				
Male	110 (47.0)	35 (58.3)	53 (43.4)	22 (42.3)
Female	124 (53.0)	25 (41.7)	69 (56.6)	30 (57.7)
Age, years; mean (SD)	63.7 (12.7)	57.6 (12.8)	64.4 (11.1)	69.1 (13.3)
< 65	114 (48.7)	41 (68.3)	54 (44.3)	19 (36.5)
65 to < 80	97 (41.5)	17 (28.3)	61 (50.0)	19 (36.5)
≥ 80	23 (9.8)	2 (3.3)	7 (5.7)	14 (26.9)
BMI, kg/m^2^; mean (SD)	22.6 (3.4)	23.4 (3.7)	22.5 (3.5)	21.9 (2.7)
Albumin, g/dL; mean (SD)	3.8 (0.6)	4.0 (0.5)	3.8 (0.6)	3.7 (0.6)
Total bilirubin, mg/dL; mean (SD)	0.6 (0.3)	0.5 (0.2)	0.6 (0.3)	0.5 (0.3)
Hemoglobin, g/dL; mean (SD)	12.5 (2.0)	13.1 (1.9)	12.5 (1.7)	11.9 (2.4)
ANC, cells/μL; mean (SD)	4176.1 (1979.6)	4147.7 (1425.1)	4246.5 (2286.0)	4043.8 (1767.7)
ALC, cells/μL; mean (SD)	1379.3 (1366.9)	1756.0 (2148.5)	1255.7 (1051.5)	1234.6 (590.1)
Relative dose intensity, %; mean (SD)	88.9 (12.4)	91.1 (13.4)	89.7 (10.8)	84.5 (14.0)
Performance status				
0–1	224 (95.7)	59 (98.3)	117 (95.9)	48 (92.3)
2–4	10 (4.3)	1 (1.7)	5 (4.1)	4 (7.7)
Diagnosis				
DLBCL	158 (67.5)	32 (53.3)	84 (68.9)	42 (80.8)
FL	40 (17.1)	16 (26.7)	20 (16.4)	4 (7.7)
Transformed DLBCL	13 (5.6)	5 (8.3)	5 (4.1)	3 (5.8)
Other^a^	23 (9.8)	7 (11.7)	13 (10.7)	3 (5.8)
Stage				
I–II	107 (45.7)	34 (56.7)	52 (42.6)	21 (40.4)
III–IV	127 (54.3)	26 (43.3)	70 (57.4)	31 (59.6)
Bone marrow involvement				
No	206 (88.0)	52 (86.7)	107 (87.7)	47 (90.4)
Yes	28 (12.0)	8 (13.3)	15 (12.3)	5 (9.6)
Complications (diabetes)				
No	213 (91.0)	53 (88.3)	111 (91.0)	49 (94.2)
Yes	21 (9.0)	7 (11.7)	11 (9.0)	3 (5.8)
Complications (liver/kidney disease)				
No	222 (94.9)	57 (95.0)	118 (96.7)	47 (90.4)
Yes	12 (5.1)	3 (5.0)	4 (3.3)	5 (9.6)
Other complications^b^				
No	110 (47.0)	29 (48.3)	61 (50.0)	20 (38.5)
Yes	124 (53.0)	31 (51.7)	61 (50.0)	32 (61.5)

Data are given as *n* (%) unless otherwise stated

*ALC* absolute lymphocyte count, *ANC* absolute neutrophil count, *BMI* body mass index, *DLBCL* diffuse large B-cell lymphoma, *FL* follicular lymphoma, *G-CSF* granulocyte colony-stimulating factor, *PS* performance status

^a^Others included mucosa-associated lymphoid tissue lymphoma, mantle cell lymphoma, and B-cell lymphoma

^b^Other complications included hypertension, hyperlipidemia, dyslipidemia, abnormal glucose tolerance, and hyperuricemia and only accounted for those that were present in ≥ 5 patients

### G-CSF selection and outcomes in cycle 1

**Fig. 1:**
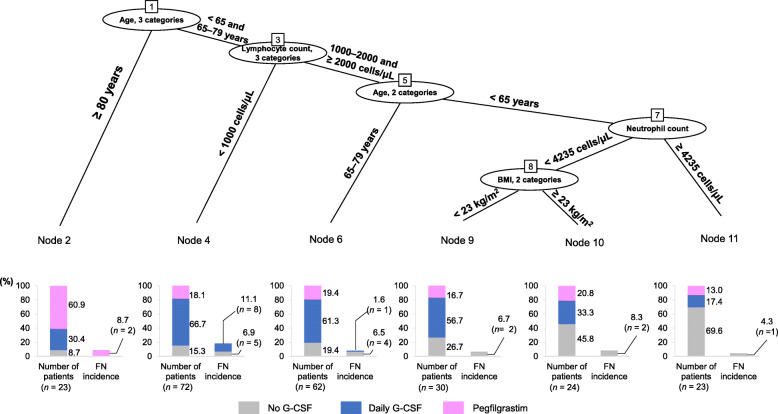
Classification tree of G-CSF, FN onset, and FN rate in cycle 1. FN incidence is shown as a percentage of the patients receiving each treatment. *BMI* body mass index, *FN* febrile neutropenia, *G-CSF* granulocyte colony-stimulating factor

As one of the specific objectives of this subanalysis was to identify the patient background factors that may aid in the selection of G-CSF preparations (pegfilgrastim or daily G-CSF), we developed a classification tree using the background factors of patients undergoing the first R-CHOP cycle as explanatory variables to predict the FN incidence rates at that time (Fig. 1**)**. The classification path of the tree analysis was as follows: age (< 65, 65–79, and ≥ 80 years), lymphocyte count (< 1000, 1000–2000, and ≥ 2000 cells/μL), age (< 65 and 65–79 years), and neutrophil count (< 4235 and ≥ 4235 cells/μL), with those who had neutrophil count < 4235 cells/μL being further categorized based on BMI (< 23 and ≥ 23 kg/m^2^). The most frequent use of pegfilgrastim was in patients aged ≥ 80 years. Conversely, the frequency of daily G-CSF use was greatest in patients aged ≤ 79 years and with lymphocyte counts of < 1000 cells/μL, and these patients were also found to have the highest FN rate. This classification tree analysis showed that the most relevant factors for selecting a G-CSF preparation in the first R-CHOP cycle were age ≥ 80 years and lymphocyte count < 1000 cells/μL, followed by neutrophil count < 4235 cells/μL and BMI < 23 kg/m^2^.

**Fig. 2:**
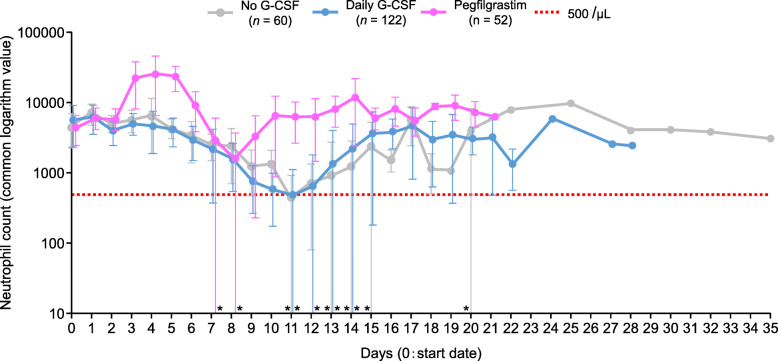
Transition of the number of neutrophils in cycle 1. *Where the standard deviation of the mean value is negative, it cannot be expressed as a common logarithmic value and the bottom of the bar is not shown. *G-CSF* granulocyte colony-stimulating factor

We evaluated whether receiving no G-CSF preparation or receiving daily G-CSF or pegfilgrastim during cycle 1 would have any effect on neutrophil count (Fig. 2). Over time, there was no notable difference in the change in neutrophil count between the no G-CSF administration and daily G-CSF groups; however, the neutrophil count began to increase relatively more rapidly after pegfilgrastim administration.

**Fig. 3:**
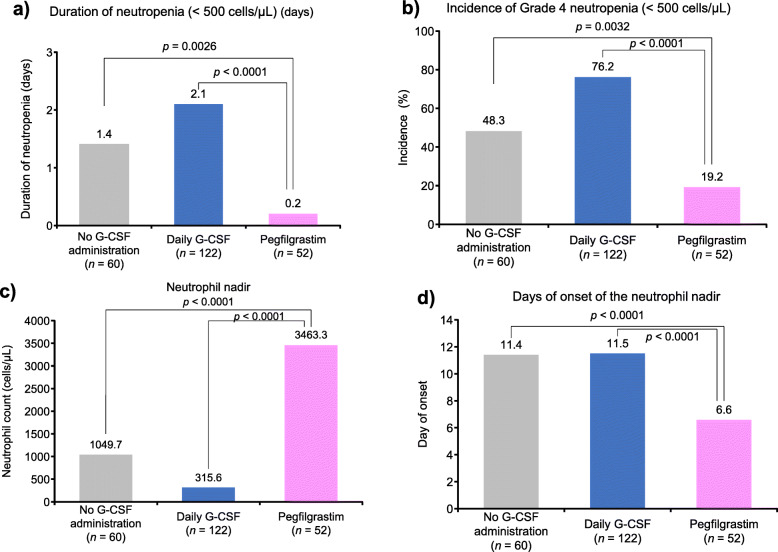
Multiple comparison of neutrophil count by G-CSF type. **a** duration of neutropenia, **b** incidence of Grade 4 neutropenia, **c** neutrophil nadir, **d** days of onset of the neutrophil nadir. *G-CSF* granulocyte colony-stimulating factor

We also compared the duration of neutropenia (< 500 cells/μL), the incidence of grade 4 neutropenia (< 500 cells/μL), lowest neutrophil count (i.e., neutrophil nadir), and day of onset of the neutrophil nadir by type of G-CSF preparation (Fig. 3**)**. Of note, the duration of neutropenia and incidence of grade 4 neutropenia were significantly higher in the no G-CSF administration group (*p* = 0.0026 and *p* = 0.0032, respectively) and the daily G-CSF group (*p* < 0.0001 for both) compared with the pegfilgrastim group. The neutrophil nadir was significantly lower in the no G-CSF group and the daily G-CSF group (*p* < 0.0001 for both) compared with the pegfilgrastim group. The day of onset of the minimum neutrophil count was significantly later for patients in the no G-CSF and daily G-CSF groups (*p* < 0.0001 for both) compared with the pegfilgrastim group. Although patients receiving pegfilgrastim had a significantly earlier onset of neutrophil nadir, they had a significantly shorter neutropenia duration, a lower incidence of grade 4 neutropenia, and a significantly higher neutrophil count nadir compared with patients treated with daily G-CSF and those who did not receive any G-CSF preparation.

### R-CHOP dose modification and G-CSF treatment in subsequent cycles

We compared the dose reductions and dose delays after cycle 2 by no administration of G-CSF preparation, daily G-CSF, and pegfilgrastim groups (Table 2). There were a total of 36 (15.4%) dose reductions after cycle 2, consisting of eight cases (13.3%) in the no G-CSF administration group, 18 cases (14.8%) in the daily G-CSF group, and 10 cases (19.2%) in the pegfilgrastim group. Overall, there were 97 (41.5%) dose delays in cycle 2: 24 (40.0%) in the no G-CSF administration group, 54 (44.3%) in the daily G-CSF group and 19 (36.5%) in the pegfilgrastim group. Of note, the use of G-CSF preparations did not seem to markedly contribute to a lesser number of dose reductions or delays compared with no use of G-CSF preparations.

**Table 2:** Dose reduction or delay after cycle 2

**First cycle**		**Reduction after cycle 2, *n*** (%)				
**G-CSF type**	***N***	**No**		**Yes**		
Overall	234	198 (84.6)		36 (15.4)		
No G-CSF administration	60	52 (86.7)		8 (13.3)		
Daily G-CSF	122	104 (85.2)		18 (14.8)		
Pegfilgrastim	52	42 (80.8)		10 (19.2)		
***Multivariate analysis*** ^***a***^***: Dose reduction*** ***Objective variable (0: no reduction, 1: dose reduction)***		**Parameter estimate**	**Standard error**	**Odds ratio**		***p*** **value**
				**OR**	**95% CI**	
FN onset in cycle 1 (absent, present)		1.7488	0.4672	5.748	2.301, 14.359	0.0002
Sex (male, female)		0.9693	0.4122	2.636	1.175, 5.914	0.0187
		**Delay after cycle 2, *n*** (%)				
		**No**		**Yes**		
Overall	234	137 (58.5)		97 (41.5)		
No G-CSF administration	60	36 (60.0)		24 (40.0)		
Daily G-CSF	122	68 (55.7)		54 (44.3)		
Pegfilgrastim	52	33 (63.5)		19 (36.5)		
***Multivariate analysis*** ^***b***^***: Dose delay*** ***Objective variable (0: absence of delay, 1: presence of delay)***		**Parameter estimate**	**Standard error**	**Odds ratio**		***p*** **value**
				**OR**	**95% CI**	
Hemoglobin (≥ 12 g/dL, < 12 g/dL)		0.8496	0.2801	2.339	1.351, 4.050	0.0024

*CI* confidence interval, *G-CSF* granulocyte colony-stimulating factor, *OR* odds ratio

^a^Variables significantly associated with dose reduction were selected (*p* = 0.05)

^b^Variables significantly associated with dose delay were selected (*p* = 0.05), the final model by stepwise selection

Another important objective was to identify potential risk factors that may lead to dose reductions and dose delays of R-CHOP therapy after cycle 2. Thus, we conducted univariate logistic regression analyses (Supplementary Table 2 (see Additional file 1)) and multivariate logistic regression analyses (Table 2) of relevant patient background factors. In univariate logistic regression for dose reduction, FN onset in cycle 1, age (≥ 65 years), female sex, hemoglobin (< 12 g/dL), and ANC (< 2690 cells/μL [1st quintile]) were identified as significant factors. As a result of the stepwise method, FN onset in cycle 1 and female sex were identified as significant risk factors for dose reduction after cycle 2. As a result of univariate logistic regression for delay after cycle 2, FN onset at cycle 1, albumin (< 3.5 g/dL), and hemoglobin (< 12 g/dL) were identified as significant factors. As a result of the stepwise method, hemoglobin (< 12 g/dL) was extracted as a risk factor for delay after cycle 2. Based on our analysis, female patients and those who present FN in cycle 1 are at significant risk of dose reductions after cycle 2. In contrast, patients with hemoglobin < 12 g/dL are at a significant risk for dose delays after cycle 2.

**Fig. 4:**
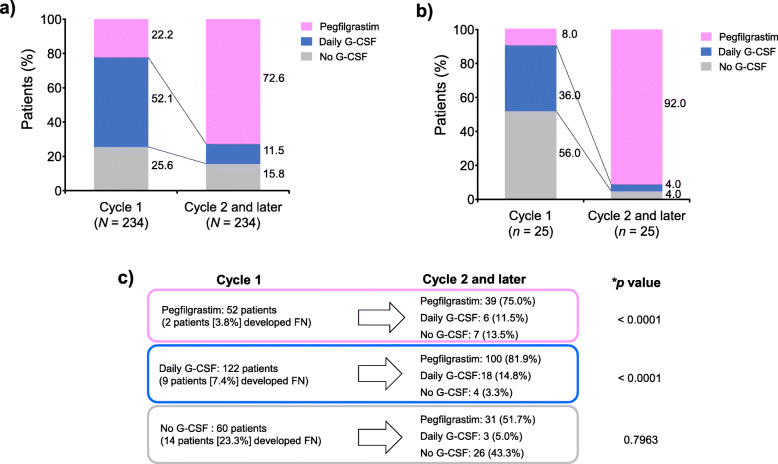
Status of G-CSF use. **a** treatment by cycle, **b** treatment by cycle in patients with FN, **c** onset of FN after cycle 2 according to the treatment received. * *p* value of the test for the null hypothesis “H0: Usage rate of pegfilgrastim after cycle 2 = 50%” versus the alternative hypothesis “H1: Usage rate of pegfilgrastim after cycle 2 ≠ 50%”. *FN* febrile neutropenia, *G-CSF* granulocyte colony-stimulating factor

Finally, we aimed to clarify the actual status of G-CSF preparation use (or no G-CSF use) after cycle 2 and onward (Fig. 4). Overall, the percentages of patients using pegfilgrastim increased markedly after cycle 2 and subsequent cycles (72.6%) from cycle 1 (22.2%). As a result, patients receiving daily G-CSF decreased by 40%. Patients who were not receiving treatment decreased by almost 10% (Fig. 4a). Among patients with FN in cycle 1, patients who received no G-CSF decreased from 56.0 to 4.0%, and those who received daily G-CSF decreased from 36.0 to 4.0% in cycle 2 and onward. Patients who received pegfilgrastim increased from 8.0 to 92.0% in cycle 2 and onward (Fig. 4b). Twenty-five of the 234 patients developed FN in cycle 1: 14 patients (23.3%) received no G-CSF; nine patients (7.4%), daily G-CSF; and two patients (3.8%), pegfilgrastim. Of patients receiving pegfilgrastim during cycle 1, 75% continued to receive pegfilgrastim during cycle 2 and onward (*p* < 0.0001). Among patients receiving daily G-CSF during cycle 1, 81.9% switched to pegfilgrastim (*p* < 0.0001), while 14.8% remained on daily G-CSF. Those who received no treatment had the highest incidence of FN (23.3%) during cycle 1, and slightly more than half switched to pegfilgrastim (51.7%; *p* = 0.7963) (Fig. 4c). Among patients with FN, 12 of 14 (85.7%) and all patients who received no G-CSF and daily G-CSF in cycle 1, respectively, switched to pegfilgrastim from cycle 2 (see Supplementary Figure 1 in Additional file 1). Of note, in cycle 2 and onward, the proportion of patients who were not receiving any G-CSF preparation decreased markedly, and even more so among patients with FN onset in cycle 1. The use of pegfilgrastim increased markedly in cycle 2 and onward, which was largely attributed to the switching of over 80% of patients (regardless of FN) receiving daily G-CSF in cycle 1 and switching of over 85% of patients who developed FN in cycle 1 but were receiving no G-CSF preparation.

## Discussion

This subanalysis of the retrospective STOP FN in NHL 2 study clarified the patient background factors that may aid in selecting a G-CSF preparation to prevent FN during chemotherapy. Additionally, we clarified possible predictors of dose reductions or delays among patients receiving R-CHOP and analyzed the current treatment patterns of G-CSF preparations in this population. These findings are relevant as these aspects of G-CSF prophylaxis have not been evaluated in Japan thus far.

In this analysis, daily G-CSF and pegfilgrastim were administered to patients primarily based on age and lymphocyte count. The more frequent use of pegfilgrastim in patients aged ≥ 80 years was likely due to clinicians adhering to the Japanese treatment guidelines [15, 16], and rates of FN in this susceptible group remained low.

Of note, older Japanese patients seemed to be receiving G-CSF preparations as recommended in the local guidelines. In contrast, studies in patients in other countries indicate that G-CSF prophylaxis was generally suboptimal [20–22], with substantial proportions of older patients failing to receive the recommended regimen [23]. In our study, the risk of developing FN appeared to be higher than that assumed by the physician. Patients aged ≤ 79 years and with a lymphocyte count of < 1000 cells/μL were most likely to have daily G-CSF administered, based on the physician’s risk assessment. Yet, patients with these factors who received daily G-CSF had a FN occurrence rate of 11.1%, compared with 6.9% for no G-CSF, and 0% for pegfilgrastim. Therefore, the likelihood of developing FN during R-CHOP treatment remains high even with daily G-CSF, which was reported to be administered later in a treatment cycle and at a lower dosage than pegfilgrastim [10]. These findings suggest the importance of carefully evaluating the FN risk to determine whether pegfilgrastim administration may be appropriate.

In this study, the lymphocyte count (< 1000 cells/μL at baseline before treatment) was more relevant than neutrophil count, according to the classification tree analysis. The lymphocyte count may be an important detail in future decision-making as, generally, the neutrophil count is the standard measure to assess G-CSF administration in current clinical practice. Other studies have also reported that the lymphocyte count was relevant in determining FN risk and was required for G-CSF administration [24, 25]. A previous paper stated that the FN rate was significantly higher among patients with lymphocyte counts < 1000 cells/μL [7]. This finding may warrant further study in prospective analyses.

In this study, patients with low BMI had a higher daily G-CSF administration rate. Lean patients and those with worse nutritional status are considered to be at a high risk of developing FN [26, 27] and may also be more susceptible to the effects of anticancer drugs. Thus, clinicians may have prescribed G-CSF more intensively for these patients.

Regarding changes in neutrophil count in cycle 1, there was no significant difference in the progression of neutrophil counts between patients who did not receive G-CSF and those who received daily G-CSF. This finding may be attributed to the administration timing. We reported that, in cycle 1, only a small percentage of patients (2.4%) started daily G-CSF treatment on days 0 to 3, with a mean of approximately 10 days for starting daily G-CSF treatment. In contrast, most patients (90.7%) treated with pegfilgrastim started treatment between days 0 and 3, with a mean of approximately 3 days for starting pegfilgrastim treatment. Further, the mean dosing period of daily G-CSF was approximately 3 days. Thus, it is possible that daily G-CSF was administered late during the cycle, or the dosing period was too short [10]. Notably, patients who received pegfilgrastim presented an increase in the neutrophil count after administration, and the neutrophil count remained high compared with the other treatments.

Although it is well known that the onset of FN during a treatment cycle can lead to chemotherapy dose delays or reductions, few studies have reported on the factors that may contribute to these dose modifications among patients with B-NHL undergoing FN prophylaxis with G-CSF. In our study, FN onset in cycle 1 and female sex were identified as risk factors for chemotherapy dose reduction, and hemoglobin (< 12 g/dL) as a risk factor for subsequent treatment delay. Taken together, current findings detailing the risk factors for FN, G-CSF treatment outcomes, and chemotherapy modification may help to inform future therapeutic decisions and improve patient health status and survival.

Additional analyses were performed to evaluate the status of G-CSF preparation use after cycle 2. Overall, the proportions of patients receiving pegfilgrastim during cycle 2 and onward increased by more than 3-fold. Among patients who developed FN in cycle 1, pegfilgrastim use increased from 8.0 to 92.0% in cycle 2 and onward. A significantly greater proportion of patients continued to use pegfilgrastim or switched from daily G-CSF to pegfilgrastim (*p* < 0.0001, each). Consequently, the proportions of patients receiving daily G-CSF and no treatment decreased considerably. In Japan, while the first cycle of chemotherapy is administered on an inpatient basis, the subsequent cycles are handled in an outpatient manner. Pegfilgrastim is administered subcutaneously using a prefilled syringe, and only needs to be administered once per cycle. Thus, the prescription of pegfilgrastim may have increased from cycle 2 because of its convenience for administration.

In contrast with the previously reported incidences of FN in cycle 1 in Japan (9.1% [7] and 10.5% [10]), the present results suggest that the incidence of FN in cycle 1 was less pronounced among patients treated with pegfilgrastim (3.8%) than daily G-CSF (7.4%) and no treatment (23.3%). These results resemble the FN incidence of 3% among patients with various tumor types who received primary prophylaxis with pegfilgrastim [28].

The main limitations of the study were the retrospective study design, in which the quality of the data was dependent on the medical record completeness, that all patients were recruited from a single center, and the possible bias arising from treatment prescription by physicians (dose and duration). However, this analysis is based on the data from the first chemotherapy cycle, which is administered on an inpatient basis in Japan. This particularity could signify accurate assessments and patient evaluations in a real-world setting.

In this analysis of Japanese patients diagnosed with B-NHL, who received at least three cycles of R-CHOP treatment, a classification tree analysis was performed to assess the use and type of prophylactic G-CSF preparation. This analysis showed that a lymphocyte count < 1000 cells/μL at baseline was associated with more frequent administration of daily G-CSF compared with pegfilgrastim, and that these patients had a high FN rate despite daily G-CSF treatment.

## Conclusions

For the first time, we have clarified the characteristics of patients based on the actual use of G-CSF preparations. The main determinants of G-CSF preparations seemed to be age and lymphocyte count. Patients aged ≥ 80 years were generally prescribed pegfilgrastim. Patients aged ≤ 79 years and with a lymphocyte count of < 1000 cells/μL were most likely to be prescribed daily G-CSF, and they tended to have higher rates of FN. Additionally, we report the predictors of R-CHOP therapy dose reductions or delays among Japanese patients with B-NHL, which were female sex, FN onset during cycle 1, and hemoglobin level. Finally, we confirmed that changes in neutrophil counts were observed when daily G-CSF or pegfilgrastim were administered and that these changes were significantly more marked with pegfilgrastim compared with daily G-CSF and without G-CSF administration. We consider that these findings have important implications on clinical decision-making, particularly when selecting the most beneficial G-CSF preparation for patients with B-NHL who are planned to undergo R-CHOP.

## Additional Files

**Additional file 1 **: **Supplementary Table 1**. Analysis settings and analysis usage variables. *BMI* body mass index, *G-CSF* granulocyte colony-stimulating factor, *PS* performance status. **Supplementary Table 2** Univariate logistic regression analyses with and without dose reduction and delay after cycle 2. *ALC* absolute lymphocyte count, *ANC* absolute neutrophil count, *BMI* body mass index, *DLBCL* diffuse large B-cell lymphoma, *CI* confidence interval, *FL* follicular lymphoma, *FN* febrile neutropenia, *G-CSF* granulocyte colony-stimulating factor, *OR* odds ratio, *PS* performance status. **Supplementary Figure 1.** Treatment received by cycles in patients who developed FN. *FN* febrile neutropenia, *G-CSF* granulocyte colony-stimulating factor.

## Data Availability

Data sharing is not applicable to this article as no new data were created or analyzed in this study.

## Abbreviations

ANC: Absolute neutrophil count
BMI: Body mass index
B-NHL: Non-Hodgkin B-cell lymphoma
FN: Febrile neutropenia
G-CSF: Granulocyte colony-stimulating factor
PS: Performance status
R-CHOP: Rituximab, cyclophosphamide, doxorubicin, vincristine, and prednisolone chemotherapy
RDI: Relative dose intensity
SD: Standard deviation
