# Supplementary material for: Factors for the optimal selection of granulocyte colony-stimulating factor preparations and predictors for R-CHOP dose reductions/delays among patients with non-Hodgkin B-cell lymphoma (STOP FN in NHL 2 subanalysis)
Source: BMC Cancer. 2021 Apr 6;21:358. doi: 10.1186/s12885-021-08068-0 (PMC8025521; doi:10.1186/s12885-021-08068-0)
Supplement: Supplementary file 1 — Additional file 1 : Supplementary Table 1. Analysis settings and analysis usage variables. BMI body mass index, G-CSF granulocyte colony-stimulating factor, PS performance status. Supplementary Table 2 Univariate logistic regression analyses with and without dose reduction and delay after cycle 2. ALC absolute lymphocyte count, ANC absolute neutrophil count, BMI body mass index, DLBCL diffuse large B-cell lymphoma, CI confidence interval, FL follicular lymphoma, FN febrile neutropenia, G-CSF granulocyte colony-stimulating factor, OR odds ratio, PS performance status. Supplementary Figure 1. Treatment received by cycles in patients who developed FN. FN febrile neutropenia, G-CSF granulocyte colony-stimulating factor. [file 12885_2021_8068_MOESM1_ESM.docx]

# Additional File 1

# Factors for the optimal selection of granulocyte colony-stimulating factor preparations and predictors for R-CHOP dose reductions/delays among patients with non-Hodgkin B-cell lymphoma (STOP FN in NHL 2 subanalysis)

Masahiro Yokoyama^1,^*, Yoshiharu Kusano^1^, Norihito Inoue^1^, Noriko Nishimura^1^, Yuko Mishima^1^, Tomoyuki Nukada^2^, Kiyohiko Hatake^3^ and Yasuhito Terui^1^

^1^Division of Hematology Oncology, The Cancer Institute Hospital, Japanese Foundation for Cancer Research, Tokyo, Japan

^2^Medical Affairs, Kyowa Kirin Co. Ltd., Tokyo, Japan

^3^Department of Hematology, International University of Health and Welfare, Tokyo, Japan

***Corresponding author**

Masahiro Yokoyama

Division of Hematology Oncology, The Cancer Institute Hospital, Japanese Foundation for Cancer Research, 3-8-31 Ariake, Koto-ku, Tokyo 135-8550, Japan

Telephone: +81-3-3520-0111

Fax: +81-3-3520-0141

Email: [masahiro.yokoyama@jfcr.or.jp](mailto:masahiro.yokoyama@jfcr.or.jp)

**Supplementary Table 1.** Analysis settings and analysis usage variables

| **(1) Analysis settings** | | | | | |
| --- | --- | --- | --- | --- | --- |
| No | | Item | | Setting | |
| 1 | | No. of patients for analysis | | 234 | |
| 2 | | Criterion variable | | G-CSF types (no G-CSF, daily G-CSF, pegfilgrastim) | |
| 3 | | Number of explanatory variables | | 14 items (quantitative variable: 1, qualitative variable: 13) of patient background (baseline). | |
| 4 | | Analysis tool | | R 3.4.1 (64 bit), Library: rpart (4.1.13) | |
| 5 | | Analysis settings | | Threshold for pruning = 0.001, number of patients ≥ 20 at the terminal node, split index = Gini Index | |
| **(2) Variables used in the analysis** | | | |  | |
| No | Classification | Variable | Attribute | Number of categories | Category |
| 1 | Criterion variable | G-CSF type | Qualitative | 3 | No G-CSF, daily G-CSF, or pegfilgrastim |
| 2 | Explanatory variable | Age (years) | Qualitative | 3 | < 65, 65–79, or ≥ 80 |
| 3 | Patients’ characteristic | BMI (kg/m^2^) | Qualitative | 2 | < 23 or ≥ 23 |
| 4 |  | Albumin (g/dL) | Qualitative | 2 | < 3.5 or ≥ 3.5 |
| 5 |  | Bilirubin (mg/dL) | Qualitative | 2 | < 1 or ≥ 1 |
| 6 |  | Hemoglobin (g/dL) | Qualitative | 2 | < 12 or ≥ 12 |
| 7 |  | Neutrophil count (cells/µL) | Quantitative | - | - |
| 8 |  | Lymphocyte count (cells/µL) | Qualitative | 3 | < 1000, 1000–2000, or ≥ 2000 |
| 9 |  | Sex | Qualitative | 2 | Male or female |
| 10 |  | PS | Qualitative | 2 | 0–1 or 2–4 |
| 11 |  | Stage | Qualitative | 2 | I–II or III–IV |
| 12 |  | Presence or absence of bone marrow involvement | Qualitative | 2 | Yes or no |
| 13 |  | Presence of complications (diabetes) | Qualitative | 2 | Yes or no |
| 14 |  | Presence of complications (liver and kidney disease) | Qualitative | 2 | Yes or no |
| 15 |  | Presence of complications (other) | Qualitative | 2 | Yes or no |

*BMI* body mass index, *G-CSF* granulocyte colony-stimulating factor, *PS* performance status

**Supplementary Table 2.** Univariate logistic regression analyses with and without dose reduction and delay after cycle 2

|  | **Parameter**  **estimate** | **Standard**  **error** | **Odds ratio** | | ***p* value** | |
| --- | --- | --- | --- | --- | --- | --- |
|  |  |  | **OR** | **95% CI** |  |  |
| ***Univariate analysis: Dose reduction***  ***Objective variable (0: no reduction, 1: dose reduction)*** | | | | | |  |
| Neutrophil count < 500/μL (0 days, 1 day) | −0.4643 | 0.5982 | 0.629 | 0.195, 2.030 | 0.4376 | |
| Neutrophil count < 500/μL (0 days, 2+ days) | 0.4802 | 0.3907 | 1.616 | 0.752, 3.476 | 0.2191 | |
| FN onset in cycle 1 (absent, present) | 1.7550 | 0.4558 | 5.783 | 2.367, 14.131 | 0.0001 | |
| Minimum neutrophil count (< 500/μL, ≥ 500/μL) | −0.2288 | 0.3706 | 0.795 | 0.385, 1.645 | 0.5369 | |
| Age (< 65 years, ≥ 65 years) | 0.9018 | 0.3888 | 2.464 | 1.150, 5.279 | 0.0204 | |
| Sex (male, female) | 0.9757 | 0.3983 | 2.653 | 1.215, 5.792 | 0.0143 | |
| PS (0–1, 2–4) | 0.3348 | 0.8121 | 1.398 | 0.285, 6.865 | 0.6802 | |
| BMI (< 23 kg/m^2^, ≥ 23 kg/m^2^) | −0.5978 | 0.3891 | 0.550 | 0.257, 1.179 | 0.1244 | |
| Disease (DLBCL, FL) | 0.2192 | 0.4733 | 1.245 | 0.492, 3.148 | 0.6433 | |
| Disease (DLBCL, Transformed DLBCL) | −13.203 | 494.7 | < 0.001 | < 0.001,  > 999.999 | 0.9787 | |
| Disease (DLBCL, Others) | 0.7283 | 0.5257 | 2.072 | 0.739, 5.805 | 0.1659 | |
| Stage (I–II, III–IV) | −0.4668 | 0.3647 | 0.627 | 0.307, 1.281 | 0.2006 | |
| Bone marrow involvement (no, yes) | 0.7102 | 0.4802 | 2.034 | 0.794, 5.215 | 0.1391 | |
| Complications (diabetes mellitus) (no, yes) | −0.5903 | 0.7666 | 0.554 | 0.123, 2.490 | 0.4413 | |
| Complications (liver/kidney disease) (no, yes) | 0.1006 | 0.7967 | 1.106 | 0.232, 5.271 | 0.8995 | |
| Albumin (≥ 3.5 g/dL, < 3.5 g/dL) | 0.5527 | 0.4028 | 1.738 | 0.789, 3.827 | 0.1700 | |
| Total bilirubin (< 1 mg/dL, ≥ 1 mg/dL) | −0.0924 | 0.7862 | 0.912 | 0.195, 4.257 | 0.9065 | |
| Hemoglobin (≥ 12 g/dL, < 12 g/dL) | 1.0087 | 0.3688 | 2.742 | 1.331, 5.649 | 0.0062 | |
| ANC (≥ 1^st^ quintile, < 1^st^ quintile; 1^st^ quintile = 2690 cells/µL) | 0.7525 | 0.4079 | 2.122 | 0.954, 4.721 | 0.0651 | |
| ALC (≥ 1^st^ quintile, < 1^st^ quintile; 1^st^ quintile = 710 cells/µL) | 0.0163 | 0.4581 | 1.016 | 0.414, 2.495 | 0.9717 | |
| ALC (≥ 1000/μL, < 1000/μL) | 0.2185 | 0.3734 | 1.244 | 0.598, 2.587 | 0.5584 | |
| G-CSF in cycle 1 (no G-CSF administration, daily G-CSF) | 0.1178 | 0.4576 | 1.125 | 0.459, 2.758 | 0.7969 | |
| G-CSF in cycle 1 (no G-CSF administration, pegfilgrastim) | 0.4369 | 0.5177 | 1.548 | 0.561, 4.270 | 0.3987 | |
|  | | | | | | |
| ***Univariate analysis: Dose delay***  ***Objective variable (0: absence of delay, 1: presence of delay)*** | | | | | | |
| Neutrophil count < 500/μL (0 days, 1 day) | −0.2433 | 0.3720 | 0.784 | 0.378, 1.626 | 0.5132 | |
| Neutrophil count < 500/μL (0 days, 2+ days) | 0.0419 | 0.2941 | 1.043 | 0.586, 1.856 | 0.8867 | |
| FN onset in cycle 1 (absent, present) | 1.0329 | 0.4402 | 2.809 | 1.186, 6.657 | 0.0189 | |
| Minimum neutrophil count (< 500/μL, ≥ 500/μL) | 0.0514 | 0.2675 | 1.053 | 0.623, 1.778 | 0.8476 | |
| Age (< 65 years, ≥ 65 years) | 0.0886 | 0.2656 | 1.093 | 0.649, 1.839 | 0.7387 | |
| Sex (male, female) | 0.1131 | 0.2661 | 1.120 | 0.665, 1.886 | 0.6709 | |
| PS (0–1, 2–4) | −0.0625 | 0.6596 | 0.939 | 0.258, 3.422 | 0.9245 | |
| BMI (< 23 kg/m^2^, ≥ 23 kg/m^2^) | −0.00277 | 0.2686 | 0.997 | 0.589, 1.688 | 0.9918 | |
| Disease (DLBCL, FL) | −0.1787 | 0.3643 | 0.836 | 0.410, 1.708 | 0.6237 | |
| Disease (DLBCL, Transformed DLBCL) | −0.4787 | 0.6222 | 0.620 | 0.183, 2.098 | 0.4417 | |
| Disease (DLBCL, Others) | 0.4191 | 0.4475 | 1.521 | 0.633, 3.656 | 0.3490 | |
| Stage (I–II, III–IV) | −0.0458 | 0.2663 | 0.955 | 0.567, 1.610 | 0.8635 | |
| Bone marrow involvement (no, yes) | 0.2301 | 0.4046 | 1.259 | 0.570, 2.782 | 0.5695 | |
| Complications (diabetes mellitus) (no, yes) | −0.1539 | 0.4703 | 0.857 | 0.341, 2.155 | 0.7436 | |
| Complications (liver/kidney disease) (no, yes) | 0.7194 | 0.6013 | 2.053 | 0.632, 6.672 | 0.2315 | |
| Albumin (≥ 3.5 g/dL, < 3.5 g/dL) | 0.8017 | 0.3215 | 2.229 | 1.187, 4.186 | 0.0126 | |
| Total bilirubin (< 1 mg/dL, ≥ 1 mg/dL) | −0.2575 | 0.5743 | 0.773 | 0.251, 2.382 | 0.6538 | |
| Hemoglobin (≥ 12g/dL, < 12 g/dL) | 0.8496 | 0.2801 | 2.339 | 1.351, 4.050 | 0.0024 | |
| ANC (≥ 1^st^ quintile, < 1^st^ quintile; 1^st^ quintile = 2690 cells/µL) | 0.0392 | 0.3360 | 1.040 | 0.538, 2.009 | 0.9070 | |
| ALC (≥ 1^st^ quintile, < 1^st^ quintile; 1^st^ quintile = 710 cells/µL) | 0.0392 | 0.3360 | 1.040 | 0.538, 2.009 | 0.9070 | |
| ALC (≥ 1000/μL, < 1000/μL) | 0.1878 | 0.2780 | 1.207 | 0.700, 2.080 | 0.4993 | |
| G-CSF in cycle 1 (no G-CSF administration, daily G-CSF) | 0.1749 | 0.3204 | 1.191 | 0.636, 2.232 | 0.5851 | |
| G-CSF in cycle 1 (no G-CSF administration, pegfilgrastim) | −0.1466 | 0.3904 | 0.864 | 0.402, 1.856 | 0.7072 | |

*ALC* absolute lymphocyte count, *ANC* absolute neutrophil count, *BMI* body mass index, *CI* confidence interval, *DLBCL* diffuse large B-cell lymphoma, *FL* follicular lymphoma, *FN* febrile neutropenia, *G-CSF* granulocyte colony-stimulating factor, *OR* odds ratio, *PS* performance status.

**Supplementary Figure 1.** Treatment received by cycles in patients who developed FN


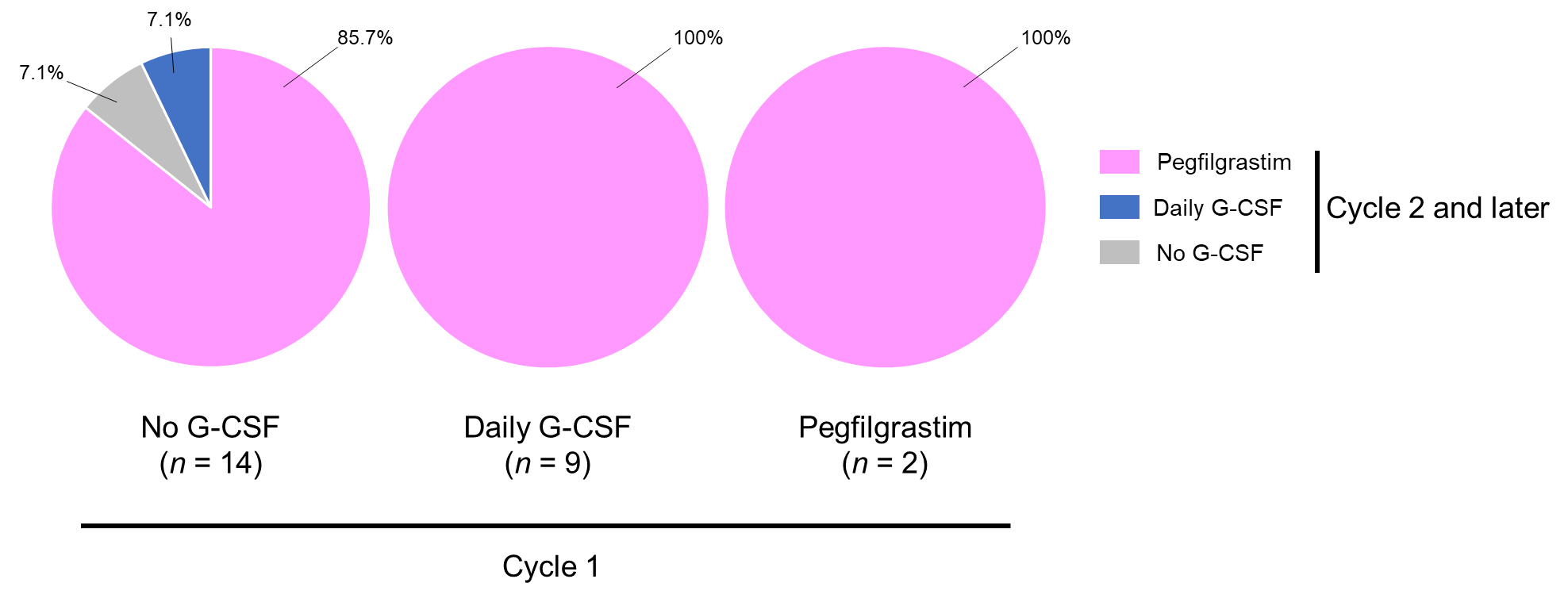


*FN* febrile neutropenia, *G-CSF* granulocyte colony-stimulating factor.
